# Helminth infections among long-term-residents and settled immigrants in Qatar in the decade from 2005 to 2014: temporal trends and varying prevalence among subjects from different regional origins

**DOI:** 10.1186/s13071-016-1433-5

**Published:** 2016-03-16

**Authors:** Marawan A. Abu-Madi, Jerzy M. Behnke, Sonia Boughattas, Asma Al-Thani, Sanjay H. Doiphode, Anand Deshmukh

**Affiliations:** Department of Biomedical Sciences, College of Health Science, Biomedical Research Center, Qatar University, P.O. Box 2713, Doha, Qatar; School of Life Sciences, University of Nottingham, University Park, Nottingham, NG7 2RD UK; Department of Laboratory Medicine and Pathology, Hamad Medical Corporation, Qatar, P.O. Box 3050, Doha, Qatar

**Keywords:** Helminths, Hookworms, *Ascaris lumbricoides*, *Trichuris trichiura*, *Hymenolepis nana*, Qatar, Immigrants, Long-term residents

## Abstract

**Background:**

Travel and migration from developing regions, where tropical diseases are common, to more developed industrialised nations can contribute to the introduction and subsequent spread of infections. With its rapidly expanding economy, Qatar has attracted vast numbers of immigrant workers in the last two decades, often from countries with poor socio-economic levels. Many used to arrive with patent intestinal parasitic infections.

**Methods:**

We analysed the prevalence of helminth infections in a dataset of 29,286 records of subjects referred for stool examination at the Hamad Medical Corporation over the course of a decade (2005 to 2014, inclusive).

**Results:**

Overall prevalence of combined helminth infections was low (1.86 %) but there were significant temporal trends, age and sex effects and those arising from the region of origin of the subjects. The most common helminths were hookworms (overall prevalence 1.22 %), which accounted for 70.1 % of cases, and therefore patterns for combined helminth infections were largely driven by hookworms. In both cases, and also in *Trichuris trichiura* and *Ascaris lumbricoides*, prevalence peaked in 2008, since when prevalence has been steadily falling. Helminth infections were largely concentrated among subjects from five Asian countries (Nepal, Bangladesh, Sri Lanka, India and Pakistan), and there was a highly biased prevalence in favour of male subjects in all cases. Prevalence of all three nematodes peaked in age class 7 (mean age 25.5 years, range = 20–29) and there were significant interactions between region of origin, sex of subjects and prevalence of hookworms.

**Conclusion:**

These results offer optimism that prevalence will continue to decline in the years ahead, especially if control is targeted at those most at risk of carrying infections.

## Background

In 2010 the number of international migrants worldwide was reported to be 214 million, representing approximately 3 % of the global population. Of those, the majority were economic migrants as well as refugees from poor, often war-torn, nations located in semi-tropical and tropical zones of the world [1]. However, travel and migration from these regions, where tropical diseases are common, can contribute to their introduction and subsequent spread into the non-endemic areas in more developed industrialised nations that attract immigrants [2]. In consequence, helminth infections in developed countries are often concentrated in impoverished immigrant communities from tropical and sub-tropical regions of sub-Saharan Africa, Asia and Latin America, living in ghettos in overcrowded inadequate housing. Poor personal hygiene practices are common and shared sanitation facilities arising from the high population densities, may also be of a very poor standard [3]. In fact, given appropriate conditions for transmission, importation of parasites by mobile populations into new geographical areas can lead to outbreaks of disease in the host country and reintroduction of infections that had previously been eradicated from those countries [4].

However, the complexity of helminth life-cycles and their geographic specificities [5, 6] can present diagnostic challenges, and they may remain undetected in non-endemic settings, which attract migrants and refugees. Local medical practitioners may be unfamiliar with the symptoms of these infections, since health care systems are often primarily focused on locally transmitted infections [7]. In the case of intestinal helminth infections, relevant public health issues are mainly concerned with the chronicity of these infections [8, 9] and the associated morbidity [10, 11]. Not surprisingly, therefore, the importation of parasitic infections by travelers [12–14] and immigrant workers into non-endemic Western countries [4, 15, 16] as well as those located in the Middle Eastern countries [17, 18], has attracted attention.

With its rapidly expanding economy, Qatar has drawn vast numbers of immigrant workers in the last two decades, often from countries with poor socio-economic levels. According to Qatar’s Ministry of Development Planning and Statistics, the total population was estimated to be 2,269,672 at the last available count on the 30^th^ November 2014. Qatari nationals number only 278,000, representing a mere 12% of the total population in the country. Indians at 545,000 and Nepalese at 400,000 actually far surpass them. Many immigrants used to arrive with patent intestinal parasitic infections, and our earlier analyses initially indicated rising trends in the prevalence of some parasitic infections [19, 20]. More recently we reported signs of a declining prevalence of helminth infections among settled immigrants [21], offering some optimism that Qatar’s current immigration procedures were having beneficial consequences in limiting the importation of parasitic diseases. Here, we build on our earlier studies on prevalence of parasitic infections up to 2011, and examine over the next three years (2012–2014) whether the declining trends have been sustained. The results of the current study will provide information on the more recent temporal trends in the prevalence of helminth infections among long-term residents and settled immigrants in the context of a dataset that spans an entire decade.

## Methods

### Study subjects and sample collection

This study was based on a retrospective survey of intestinal parasitic infections based on the records held at Hamad Medical Corporation (HMC) data-base (MediCom) maintained at the Department of Laboratory Medicine and Pathology at HMC and its outpatient clinics between 2005 and 2014.We examined the records of patients referred to different departments of the HMC hospitals including maternity, paediatrics, internal medicine and gastroenterology, and who participated in a routine stool test. Such subjects have been resident in Qatar for at least four years, a requirement for eligibility for treatment at HMC.

After removing duplicate records for subjects registered in the period 1^st^ January 2012 to 31^st^ December 2014, and retaining only first records for those with multiple entries in this period, we combined these data with two previously analysed dataset for the periods from 2005 to 2008, and 2009 to 2011. From the resulting 33,665 records, we removed 1951 records of children from 2 days to less than 7 months of age in order not to bias long-term trends in our analysis, since the earlier analysis from 2005 to 2008 had been confined to those over 7 months of age. Among this group there were no cases of helminth infections.

A further 706 records comprising subjects from Europe (*n* = 338), North America (*n* = 310), Central America (*n* = 11), Australia (*n* = 25), South America (*n* = 19) were also removed, because these continents had not been considered in our earlier analyses. Three more records were removed because of unknown nationality of the subjects. Among these 706 subjects none were infected with helminths. Next, 1565 records were removed because they were second assessments in periods 2 or 3 of individual patients seen previously in either period 1 or 2 (and hence replication of subjects), and finally 157 records were excluded because they represented a third record of subjects for which there were records in each of the three periods. Therefore, the final database only included the initial records and comprised 29,286 subjects from 69 countries. The wide range of nationalities of resident workers is consistent with Qatar’s policy for encouraging visiting and eventually resident workers from an increasingly broad range of countries.

### Ethical approval

Ethical approval for access to these data was obtained from the Medical Research Centre and Research Committee at HMC, Qatar (Research protocol # 11110/11(NPRP 4-1283-3-327). Each subject was given a specific reference number when presenting at the MHC for the first time. Age, sex and geographical region, along with other personal information that are required for clinical assessment and subsequent treatment were all recorded for each patient prior to taking the specimen and maintained on the HMC database. We downloaded only the ID reference number (necessary to eliminate repeated entry, as for example on second and subsequent presentation at the hospital) along with birth dates and treatment dates (used to calculate age at assessment), year, sex, country of origin and parasite data. Thus confidentiality was maintained throughout and the identity of subjects was not available to us, other than through each individual’s reference number.

### Stool examination

Faecal samples were obtained from subjects referred for examination at HMC as part of a routine screening policy for the diagnosis of diseases associated with intestinal infections. Stools were collected and processed as described earlier [19].

### Definition of variables

All birth dates and examination dates were recorded meticulously and the ages of subjects were classified into ranges by years. Thirteen age classes were then constructed to span ≤1 year, 1.1–1.9, 2.0–4.9, 5.0–9.9, 10.0–14.9, 15.0–19.9, 20.0–29.9, 30.0–39.9, 40.0–49.9, 50.0–59.9, 60.0–69.9, 70.0–79.9, and < 79.9 years.

The subjects in this study came from 69 countries. For the purpose of analysis, the subjects were grouped into four geographical groups for comparison with Qatari nationals (*n* = 9357). These were as follows: (i) from six countries in the Arabian Peninsula (*n* = 1441, Kuwait, Bahrain, Oman, Saudi Arabia, United Arab Emirates and Yemen); (ii) from seven countries in the Eastern Mediterranean (*n* = 2799, Iraq, Jordan, Lebanon, Palestine, Syria and Turkey); (iii) from 31 countries in Africa (*n* = 5354, Algeria, Benin, Burkina Faso, Cameroon, Chad, Comoros, Djibouti, Egypt, Eritrea, Ethiopia, Gambia, Ghana, Guinea, Kenya, Liberia, Libya, Malawi, Mali, Mauritania, Mauritius, Morocco, Mozambique, Niger, Nigeria, Senegal, Somalia, South Africa, Sudan, Tanzania, Tunisia and Uganda); and (iv) from 25 countries in Asia (*n* = 10,335, Afghanistan, Azerbaijan, Bangladesh, Bhutan, Burma, China, India, Indonesia, Iran, Japan, North Korea, South Korea, Kyrgyzstan, Malaysia, Maldives, Nepal, Pakistan, Philippines, Singapore, Sri Lanka, Tajikistan, Thailand, Turkmenistan, Uzbekistan and Vietnam). Note that unlike in our earlier two papers [20, 21], Kuwait was classified here as part of the Arabian Peninsula.

The analysis was based on data recorded at the Department of Laboratory Medicine and Pathology, HMC from 1^st^ January 2005 until 31^st^ December 2014, and is coded by year of study. However, since an analysis has already been published for the period 2005–2008 [20], and for a comparison of 2005–2008 with 2009–2011 [21], we call these periods 1 and 2 respectively, and in some analyses compare prevalence rates in these periods with period 3 covering the years 2012–2014. In our previous works [20, 21] while we had ensured there was no duplication of records (i.e. patients being assessed on more than one occasion) within each period, in [21] subjects that had first reported in period 1 and were seen again in period 2 were not removed (*n* = 596), hence some of the values in the current report for period 2 will differ marginally from those given in [21].

### Statistical analysis

Briefly, the statistical analysis was carried on data recorded at HMC database when subjects first presented at the hospital, all subsequent records having been eliminated after identification using the individual reference numbers of subjects. Prevalence data (percentage of subjects infected) are shown with 95 % confidence limits (CL_95_), calculated as described in [22] employing bespoke software. In some figures we have omitted confidence levels so as not to obscure trends in the illustrated data (but these are available from the authors on request). Prevalence was analysed by maximum likelihood techniques based on log-linear analysis of contingency tables using the software package SPSS (Version 22.0.0). Initially, full factorial models were fitted, incorporating as factors SEX (2 levels, males and females), AGE (13 levels), YEAR of study (10 levels, for each of the years from 2005 to 2014) and REGION of origin (5 levels, Africa, Arabian Peninsula, Asia, Eastern Mediterranean and Qatar). In some analyses PERIOD was fitted rather than YEAR because we wanted to know whether prevalence had changed between the first period (2005–2008), second (2009–2011) and third (2012–2014) periods. The presence/absence of a parasite or parasites was considered as a binary factor and is referred to as INFECTION in the analysis. These explanatory factors were fitted initially to all models that were evaluated, and these were then reduced by backward selection to derive a minimum sufficient model in which the importance of each term (i.e. interactions involving infection) was assessed by the probability that its exclusion would alter the model significantly as described previously [21].

## Results

Of the 29,286 subjects who fulfilled the inclusion criteria, 544 (1.86 %; CL_95 =_ 1.703–2.012) were infected with helminths, although it should be noted that this figure is an under-estimate because in Period 1 (2005–2008) the only helminths that were recorded were hookworms, *Trichuris trichiura*, *Ascaris lumbricoides* and *Hymenolepis nana*. The overall prevalence of each of the species over the ten year period of the study is given in Table 1, and prevalence is given also for the first, second and third periods. These data show that there was a continuing trend of falling prevalence for each of the nematode species from Period 1 to Period 3.

**Table 1:** Prevalence (%) of helminth parasites in the study population in the first (2005–2008), second (2009–2011), third (2012–2014) periods and overall

		Prevalence (95 % CL)		
	Period 1 2005–2008 (*n* = 9,208)	Period 2 2009–2011 (*n* = 8,844)	Period 3 2012–2014 (*n* = 11,234)	Combined^d^ (*n* = 29,286)
Four common species				
Hook worms	2.05 (1.763–2.342)	1.38 (1.136–1.623)	0.42 (0.307–0.556)	1.22 (1.097–1.348)
*Trichuris trichiura*	0.49 (0.356–0.654)	0.42 (0.295–0.577)	0.13 (0.075–0.220)	0.33 (0.269–0.404)
*Ascaris lumbricoides*	0.34 (0.229–0.478)	0.19 (0.112–0.308)	0.12 (0.068–0.209)	0.21 (0.162–0.271)
*Hymenolepis nana* ^a^	0.10 (0.045–0.186)	0.19 (0.112–0.308)	0.15 (0.088–0.242)	0.15 (0.106–0.198)
Above 4 helminth spp. combined	2.63 (2.301–2.955)	2.01 (1.720–2.305)	0.76 (0.604–0.936)	1.72 (1.575–1.873)
Other species				
*Strongyloides* sp.	Nd	0.16 (0.087–0.266)	0.04 (0.014–0.104)	0.09 (0.057–0.148)
*Schistosoma* sp.	Nd	0.06 (0.018–0.132)	0.07 (0.031–0.140)	0.06 (0.034–0.111)
Taenia sp.	Nd	0.02 (0.003–0.082)	0.04 (0.010–0.091)	0.03 (0.011–0.065)
Others combined^b^	Nd	0.03 (0.007–0.099)	0.04 (0.014–0.104)	0.04 (0.017–0.079)
All helminths combined^c^	Nd	2.23 (1.920–2.535)	0.93 (0.757–1.113)	1.50 (1.336–1.672)

^a^This species is also known as *Rodentolepis nana*

^b^This row includes 8 cases of three rarely encountered species*, Enterobius vermicularis*, *Fasciola hepatica* and *Trichostrongylus* sp

^c^This row summarises data for periods 2 and 3, but does not include period 1 when some of the helminths were not recorded

^d^Overall prevalence across periods 1, 2 and 3 combined or periods 2 and 3 combined when relevant data for period 1 were not available

Nd = not done, these species were not assessed independently in the first period

### Temporal changes

**Fig. 1:**
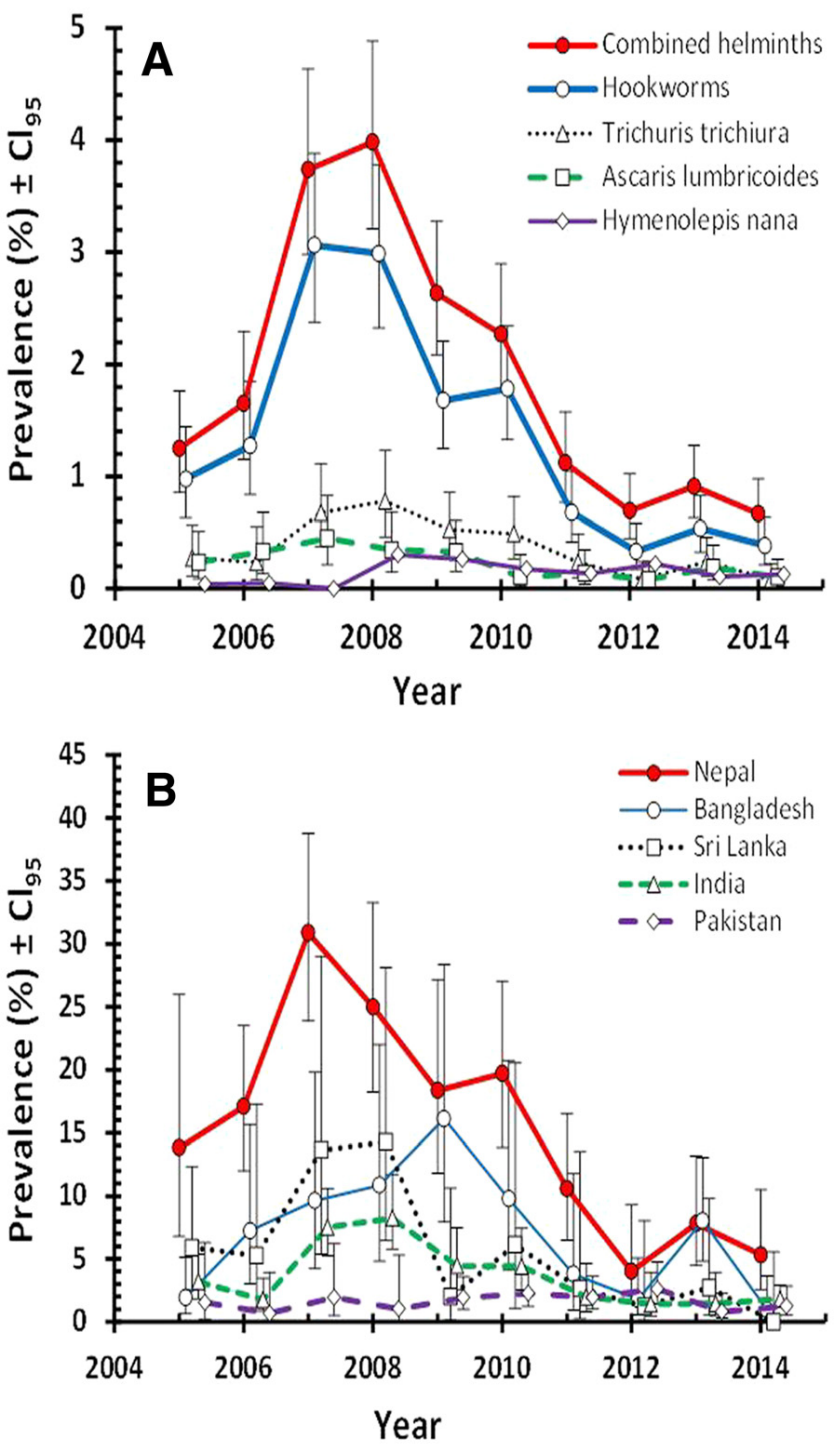
Temporal effects on the prevalence of helminths. **a** Temporal changes in prevalence of each of the four species and their combined data detected across the decade. The number of subjects included in each of the years between 2005 and 2014 inclusive, was as follows: 2559; 2120; 2220; 2309; 3038; 2860; 2945; 3604; 3728; and 3902, respectively; **b** Temporal changes in prevalence of combined helminths among subjects from the five Asian countries subset. The number of subjects in these five countries was as follows: Nepal, *n* = 1429; Bangladesh, *n* = 945; Sri Lanka, *n* = 534; India, *n* = 3571; and Pakistan, *n* = 2087

Four species of helminth (hookworms, *T. trichiura*, *A. lumbricoides* and *H. nana*) were recorded across the whole of the ten year period (Table 1). The effect of YEAR (Fig. 1a) was significant for combined helminths (*χ*^2^_9_ = 181.3.0, *P* < 0.001), hookworms (*χ*^2^_9_ = 174.6, *P* < 0.001), *T. trichiura* (*χ*^2^_9_ = 38.2, *P* < 0.001), *A. lumbricoides* (*χ*^2^_9_ = 17.2, *P* = 0.046) and *H. nana* (*χ*^2^_9_ = 17.1, *P* = 0.047).

The data in Fig. 1 show that combined helminth prevalence peaked in 2007–2008; this was largely attributable to hookworm infections, although a higher prevalence of *T. trichiura* was also evident in that period. In the following years prevalence of both hookworms and *T. trichiura* fell, reaching a low in 2012, which was then maintained over the following two years. Infections with *A. lumbricoides* and *H. nana* were rarer, but even among these two species peak prevalence was recorded in 2008 (0.35 and 0.30 %, respectively), followed by a gradual decline thereafter with the lowest prevalence of *A. lumbricoides* in 2012 (0.08 %). Despite the generally lower values for prevalence of this species, over successive years, prevalence nevertheless followed broadly the same pattern as in hookworms and *T. trichiura* (Fig. 1a). Prevalence of *H. nana* also fell after 2008, but the lowest recorded prevalence was earlier in 2007, when no tapeworm infections were detected, so prevalence was numerically higher in Period 3 compared with Period 1, as stated above.

### Region of origin of subjects

In all four species and when combined, there was a highly significant effect of REGION (Table 2; all four species combined, *χ*^2^_4_ = 432.4, *P* < 0.001; hookworms *χ*^2^_4_ = 666.3, *P* < 0.001; *T. trichiura χ*^2^_4_ = 108.5, *P* < 0.001; *A. lumbricoides χ*^2^_4_ = 88.8, *P* < 0.001; *H. nana χ*^2^_4_ = 39.7, *P* < 0.001). No helminth infections at all were detected among subjects from the Eastern Mediterranean and there were only five cases among those from the Arabian Peninsula and four among the Qataris. Helminth infections were largely concentrated among the Asian (*n* = 476; 94 % of all helminth cases) and African (*n* = 20; 4.0 % of all helminth cases) subjects.

**Table 2:** Number of subjects in each category and the prevalence (%) of the four species of helminth by sex, and region of origin

	No. of subjects	Hookworms	*T. trichiura*	*A. lumbricoides*	*H. nana*	Combined
Host sex						
Males	16,991	***2.01***	***0.49***	***0.30***	***0.19***	***2.69***
Females	12,295	0.14	0.11	0.09	0.09	0.39
Region						
Arabian Pen.	1,441	0	0	0	***0.35***	0.35
Eastern Med.	2,799	0	0	0	0	0
Africa	5,354	0.11	0.06	0.04	0.19	0.37
Asia	10,335	***3.38***	***0.91***	***0.58***	0.26	***4.61***
Qatar	9,357	0.03	0	0	0.01	0.04

The highest prevalence in each category is in bold italics for emphasis

Statistical outputs were derived from minimum sufficient models, after first fitting for each species in turn, all variables into a single full factorial model, and then stepwise backward deletion of non-significant terms. The *χ* ^2^ values for goodness of fit of the minimum sufficient models for hookworms, *T. trichiura*, *A. lumbricoides*, *H. nana* and all helminths combined was as follows: 792.2 (df = 1854, *P* = 1), 717.9 (df =1813, *P* = 1), 720.4 (df = 1814, *P* = 1), 759.6 (df = 1814, *P* = 1) and 978.7 (df = 1846, *P* = 1), respectively. The importance of each factor in the final minimum sufficient model for each taxon is given in the text

We next explored prevalence among the individuals from the different Asian and African countries. There were no cases of helminth infection among 13 Asian countries, all being confined to the remaining 12. Among the eight countries where the sample size was more than 100 subjects, the highest prevalence was recorded among the Nepalese (15.26 %; Cl_95_ = 13.391–17.120), then in descending order among those from Bangladesh (6.8 %), Sri Lanka (4.5 %), India (3.3 %), Pakistan (1.6 %), Indonesia (0.9 %), Philippines (0.8 %) and Iran (0.4 %).

Because the highest prevalence levels among Asians were among subjects from the five Asian countries, we next investigated how prevalence among subjects from these countries had changed over the decade (Fig. 1b). In the period 2005 until 2013, prevalence was clearly higher among the Nepalese compared to any of the other nationalities. However, as prevalence declined among nationals from all five countries, towards the end of this decade, the difference between these national groups became marginal.

Among immigrants from 31 African countries, those from 27 countries (*n* = 721) were not infected, all 20 cases being confined to just four countries: Ethiopians (prevalence = 3.89 %; Cl_95_ = 1.419–9.255; *n* = 180); Egyptians (prevalence = 0.32 %; Cl_95_ = 0.155–0.593; *n* = 3102); Sudanese (prevalence = 0.15 %; Cl_95_ = 0.018–0.536; *n* = 1347) and one case among the four Gambians.

### Age of subjects

**Fig. 2:**
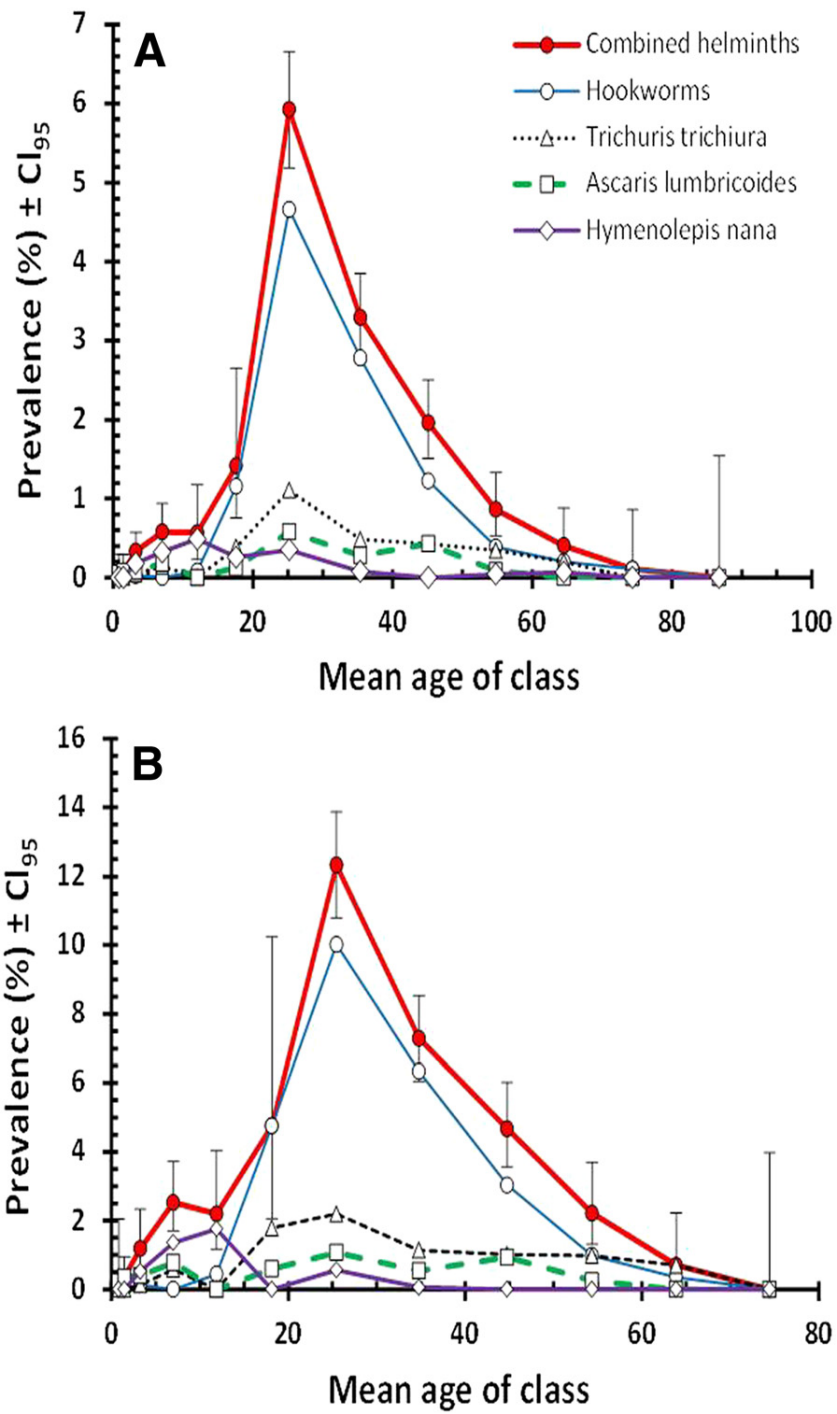
Age-prevalence profile for combined helminths, hookworms, *Trichuris trichiura*, *Ascaris lumbricoides* and *Hymenolepis nana* in the complete dataset (*n* = 29,286) (**a**) and in subjects from the five Asian countries subset (Bangladesh, India, Nepal, Pakistan and Sri Lanka), among whom most of the helminths were detected (*n* = 8566) (**b**). Legend in B as in A

For all four species combined and for each of the individual species (Fig. 2a), there was a significant effect of host age (all four species combined, *χ*^2^_12_ = 24.3, *P* = 0.018; hookworms *χ*^2^_12_ = 320.3, *P* < 0.001; *T. trichiura χ*^2^_12_ = 51.6, *P* < 0.001; *A. lumbricoides χ*^2^_12_ = 24.6, *P* = 0.017; *H. nana χ*^2^_12_ = 52.8, *P* < 0.001). However, since most of the helminth infections were detected among subjects from five Asian countries (94.3 %) we also examined the age-prevalence profile for this subset (Fig. 2b). Peak prevalence was in age class 7 in both the complete dataset (mean age = 25.2 years) and in the five Asian countries subset (mean age = 25.5 years), with prevalence values double those in the latter dataset on exclusion of regions and nationalities showing no or very low prevalence of helminths. The prevalence of *T. trichiura* and *A. lumbricoides* also peaked in this age class, but not that of *H. nana* which showed peak prevalence among younger subjects in age class five (for the five Asian countries, mean age = 11.9 years).

### Sex of subjects

Prevalence values for male and female subjects are summarised in Table 2. Male subjects harboured 90.5 % (Cl_95 =_ 88.51–92.21) of the combined helminth infections, prevalence being 6.8 times higher among males, and this difference between the sexes was significant (*χ*^2^_1_ = 267.55, *P* < 0.001). For hookworm infections, the discrepancy between the sexes was even greater (*χ*^2^_1_ = 266.5, *P* < 0.001). Prevalence was 14.4 times higher among males, who accounted for 95.3 % of all hookworm infections. There were only 97 cases of *T. trichiura* and again, most were harboured by male subjects (85.6 % of all *T. trichiura* positive cases were among male subjects) among whom prevalence was 4.5 times higher than among females (Table 2; *χ*^2^_1_ = 4.8, *P* = 0.029). There were even fewer cases of *A. lumbricoides* (*n* = 62), but for this species also most infections were detected among male subjects (82.3 % of all *A. lumbricoides* positive cases were among male subjects) and prevalence was 3.3 times higher among males (Table 2). The dichotomy between the sexes was significant when tested alone (*χ*^2^_1_ = 16.7, *P* < 0.001), but not when REGION, AGE and YEAR were included as factors, suggesting that it arose through the confounding effects of other factors. Finally, infections with *H. nana* also showed male bias, since 74.4 % of cases were among males and prevalence was 2.1 times higher among males, but as above, although this difference in prevalence between the sexes was significant only when tested alone (*χ*^2^_1_ = 5.04, *P* = 0.025), it was not when other factors were included in the model.

### The effect of interactions between factors affecting prevalence

**Fig. 3:**
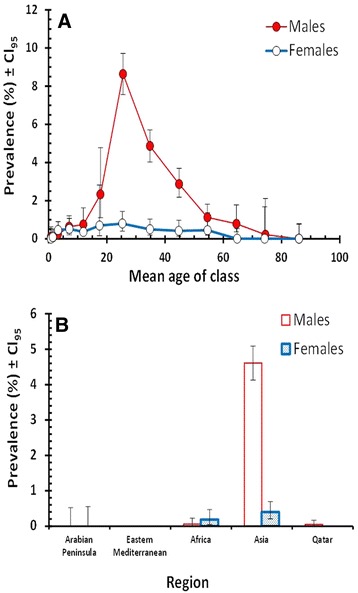
The effect of host sex on prevalence of helminths. **a** Age prevalence profiles for combined helminths in male and female subjects; **b** Prevalence of hookworms among male and female subjects from the five regions in the study

Significant interactions affecting INFCTION were detected only between AGE and SEX in the case of combined helminths (*χ*^2^_12_ = 28.8, *P* = 0.004). This is illustrated in Fig. 3a, where it can be seen that prevalence of helminths was very similar in both sexes among the three youngest age classes but then stayed relatively low among older females, whilst increasing markedly in males to peak in age class 7 before falling in the older male age classes. Since hookworms accounted for most of the helminths, this pattern was largely attributable to hookworm infections (data not shown).

Figure 3b shows the interaction of REGION and SEX with hookworms (*χ*^2^_4_ = 13.7, *P* = 0.008). The marked disparity between the sexes is clearly evident among the Asian subjects where infections dominate among male subjects, but among subjects from Africa prevalence was biased in favour of females (males = 0.06 %; CL_95_ = 0.008–0.228, *n* = 3172; females = 0.18 %; CL_95_ = 0.050–0.469, *n* = 2182).

### Combinations of helminths

Restricting the analysis to the four helminth species that were recorded in each of the three periods, there were 28,781 uninfected subjects, 454 subjects with just one species of helminth, 47 with two species, four with three species and none with all four. Based on overall prevalence figures [23] predict that in the absence of interactions between species, and based only on prevalence values for each species in turn, there should be 28,730; 554; 3; zero and zero subjects with no, one, two, three and four species respectively. This represents a significant difference to our data (*χ*^2^ = 802.8, *P* < 0.0001), implying that some helminth infections were aggregated in subsets of the data. Consistent with this prediction, all four cases of triple species infection were among subjects from the five Asian countries subset, as were 46 of the 47 double species infections. Among the 47 double species infections there were 20 cases of hookworms + *T. trichiura*, 13 of hookworms + *A. lumbricoides*, 12 of *A. lumbricoides* + *T. trichiura* and two of hookworms + *H. nana*. Among the triple species infections there were three cases of hookworms + *A. lumbricoides* + *T. trichiura* and one of hookworms + *T. trichiura* + *H. nana.*

## Discussion

In this paper we have built on our earlier published studies and shown that the declining prevalence of helminth infection, which we first noted in 2009 [21], has been largely sustained in recent years. Compared to the peak years 2007 and 2008 [20], helminth infections have continued to decline among long-term residents and settled immigrants in Qatar. In the most recent period (2012–2014), the prevalence of helminth infections has fallen to levels well below those recorded in 2005 when our long-term monitoring began, especially in the case of hookworms and *A. lumbricoides*. It should be noted however, that in Period 1 (2005–2008) the only helminths that were recorded were hookworms, *T. trichiura*, *A. lumbricoides* and *H. nana*. Although the overall prevalence of helminths was generally low, immigrants from some countries were particularly prone to carrying worms despite their status as residents in Qatar.

Among subjects from the five Asian countries subset, which we had identified in earlier papers as those most likely to carry helminth infections [20, 21], prevalence has declined substantially. Although Nepalese immigrants showed the highest prevalence of soil-transmitted helminths (STH) of all (when considered across the whole of the decade), followed by Bangladeshi and Sri Lankan immigrants, and especially so in the peak years, the distinction between immigrant workers from these countries was not nearly as marked in 2012–2014. In the latter period, only the Nepalese still stood out as showing higher prevalence than the other nationals and there was little difference between immigrants from the other Asian countries. As we have explained earlier [20], in some respects this is not surprising given that the Nepalese workers constitute the largest immigrant work force in Qatar (Medical Commission database) and given the poverty associated with the country and the documented prevalence of helminth infections among rural communities in Nepal [24].

As in our earlier reports, helminth infections were largely carried by male subjects, especially those in age class 7 (mean age of 25.6, range 20–29.9 years of age). These are most likely to be the construction workers who are largely recruited from among the poorer unskilled sectors of the communities in their countries of origin and work as labourers on the building projects in Doha City. Unfortunately, it was not possible to fit each subject’s occupation into our statistical models because relevant data were not available to us in the HMC Database. Presumably many of these workers came from villages where transmission of STH is known to occur [25]. Similar observations have been reported also for young male labourers in Taiwan [26] and Thailand [11], where helminth infections were attributed to poverty, low educational levels and geographical isolation of the villages from which the labour force was recruited [27–29]. Therefore, while young males from Nepal, were the principal carriers of helminth infections, even among these prevalence has dropped remarkably in recent years.

In the past, we have considered that it is highly unlikely that transmission of STH actually occurs frequently in Qatar, although feline hookworms (*Ancylostoma tubiaeformae*) are known to affect the cat population in Doha City [30]. Thus the possibility that transmission of human hookworms can take place locally cannot be eliminated entirely, and it may be that some transmission does occur among the long-term residents living in labour camps where conditions may be cramped and sanitation facilities stretched [20]. Infection with hookworm larvae is mainly *via* penetration of unprotected skin. According to the native settings in their countries of origin, transmission of hookworms occurs when walking barefoot or sitting on faecally contaminated soil or sand, behavioral traits that are frequently observed in rural communities in Asian and African countries [6, 31]. However, transmission *via* these routes is unlikely in the urban environment in Doha City, where pavements and open air recreational areas such as parks, are generally kept clean. Contamination of the environment by human excreta is rare, other than possibly in some of the worst of the living quarters of the labourers where conditions may be less than desirable.

In contrast to hookworm infections, both *A. lumbricoides* and *T. trichiura* are transmitted *via* embryonated eggs containing infective larvae and infection occurs when these find their way into food or drinking water, hence transmission is *via* the faecal oral route [5]. Our records revealed very few cases of *A. lumbricoides* and *T. trichiura* in Doha among long-term residents and the few infections that were detected in our data are most likely to have been imported upon original arrival of the immigrants or during home visits [12].

Intestinal helminths are renowned for their chronicity, especially the hookworm *Necator americanus* which have been shown to survive in subjects that were not re-infected for 17–18 years [8, 9]. In our view, rather than being acquired in Qatar, it is most likely that these infections were long-term, persisting from the initial arrival of the subjects in Qatar as a result of drug failure [32, 33] or failure to be treated on or just before arrival in Qatar. Home visits undertaken during long-term employment may also have exacerbated infection levels [12], rather than the infections being acquired in Qatar itself, and may not have been detected on return to Qatar.

The most exciting and encouraging finding of the current work is that the prevalence of helminth infections among long-term residents and settled immigrants has fallen greatly in the last five years. Changes in the laws governing health inspection and associated treatments at immigration to Qatar have probably played a crucial role in curtailing importation of helminths. Indeed as reported previously, it is now mandatory for all applicants for jobs in Qatar to have pre-employment certificates (PEC), which are provided at local Qatar embassy-approved clinical centres in the countries of origin before arrival in Qatar, and require medical examinations that include faecal examination. Before obtaining a certificate, subjects who are found to be positive for parasites, are required to have treatment (usually albendazole for helminths and metronidazole for protozoans) and then followed up with re-examination. The PEC was introduced six years ago in 2009, and is particularly enforced by Qatar Embassy officials when providing visas for prospective workers. Any applicant with persistent infection will not be granted a visa for entry to Qatar. Therefore, the declining prevalence of helminth infections in the last five years may be attributable in part to this initiative by the Qatar Public Health Authorities, and our data provide encouraging support for the success of this policy [21]. However, this procedure is not applicable when immigrants travel back to their countries of origin for visits and then return to Qatar to continue their employment. Falling helminth levels in Qatar may also have stemmed partly from recent helminth control and deworming programs in Asia and globally, with the preventive chemotherapy installation supported by the WHO [34–38].

## Conclusions

In conclusion, as we reported previously [20, 21], the helminth infections in our study were largely carried by Asian immigrants and were probably mostly acquired abroad either persisting from before their original arrival in Qatar or acquired subsequently on return visits to their homelands. This update of current trends should provide the health authorities in Qatar with crucial information to consolidate current health inspection procedures for immigrants. Our results should help to refine further the implementation of control programs for intestinal helminth infections in the country in order to ensure the continuing decline and eventually eradication of these infections among the inhabitants of Qatar, and the effects of their associated morbidity among the labor force.

## References

[1] World Migration Report 2013 https://publications.iom.int/fr/books/world-migration-report-2013.

[2] Gualdieri L, Rinaldi L, Petrullo L, Morgoglione ME, Maurelli MP (2011). Intestinal parasites in immigrants in the city of Naples (southern Italy). Acta Trop.

[3] Marieke H, Cumming O, Peletz R, Chan GKS, Brown J, Baker K (2014). Shared sanitation versus individual household latrines: a systematic review of health outcome. PLoS One.

[4] Monge-Maillo B, Lopez-Velez R, Norman FF, Ferrere-Gonzalez F, Martınez-Perez A, Perez-Molina JA (2015). Screening of imported infectious diseases among asymptomatic Sub-Saharan African and Latin American immigrants: a public health challenge. Am J Trop Med Hyg.

[5] Brooker A, Clements ACA, Bundy DAP (2006). Global epidemiology, ecology and control of soil-transmitted helminth infections. Adv Parasitol.

[6] Hotez PJ, Bethony J, Bottazzi ME, Brooker S, Buss P (2005). Hookworm: “the great infection of mankind”. PLoS Med.

[7] Abbas A, Newsholme W (2009). Diagnosis and recommended treatment of helminth infections. Prescriber.

[8] Palmer ED (1955). Course of egg output over a 15 year period in a case of experimentally induced *necatoriasis americanus*, in the absence of hyperinfection. Am J Trop Med Hyg.

[9] Beaver PC (1988). Light, long-lasting *Necator* infection in a volunteer. Am J Trop Med Hyg.

[10] Roche M, Layrisse M (1966). The nature and causes of hookworm anemia. Am J Trop Med Hyg.

[11] Kaewpitoon SJ, Loyd RA, Kaewpitoon N (2015). A cross-sectional survey of intestinal helminthiases in rural communities of nakhon ratchasima province, Thailand. J Med Assoc Thai.

[12] Monge-Maillo B, Norman FF, Perez-Molina JA, Navarro M, Dıaz-Menendez M, Lopez-Vélez R (2014). Travelers visiting friends and relatives (VFR) and imported infectious disease: travelers, immigrants or both? a comparative analysis. Travel Med Infect Dis.

[13] Showler AJ, Wilson ME, Kain KC, Boggild AK (2014). Parasitic diseases in travelers: a focus on therapy. Expert Rev Anti Infect Ther.

[14] Soonawala D, van Lieshout L, den Boer MA, Claas EC, Verweij JJ, Godkewitsch A (2014). Post-travel screening of asymptomatic long-term travelers to the tropics for intestinal parasites using molecular diagnostics. Am J Trop Med Hyg.

[15] Cuenca-Gómez JA, Salas-Coronas J, Cabezas-Fernández MT, Vázquez-Villegas J, Soriano-Pérez MJ, Cobo F (2013). Imported hookworm infection in Almeria. Enferm Infecc Microbiol Clin.

[16] Calderaro A, Montecchini S, Rossi S, Gorrini C, De Conto F, Medici MC (2014). Intestinal parasitoses in a tertiary-care hospital located in a non-endemic setting during 2006–2010. BMC Infect Dis.

[17] Alkarmi T, Alharbi S, Abu-Lisan M, Salman A, Behbehani K (1991). Prevalence of intestinal parasitic infections in Kuwait. Med Princ Pract.

[18] Taha HA, Soliman MI, Banjar SA (2013). Intestinal parasitic infections among expatriate workers in Al-madina Al-munawarah, kingdom of Saudi Arabia. Trop Biomed.

[19] Abu-Madi MA, Behnke JM, Ismail A (2008). Patterns of infection with intestinal parasites in Qatar among food handlers and housemaids from different geographical regions or origin. Acta Trop.

[20] Abu-Madi MA, Behnke JM, Doiphode SH (2010). Changing trends in intestinal parasitic infections among long-term-residents and settled immigrants in Qatar. Parasit Vectors.

[21] Abu-Madi MA, Behnke JM, Doiphode SH (2013). Intestinal parasitic infections among long-term-residents and settled immigrants in Qatar in the period 2005 to 2011. Am J Trop Med Hyg.

[22] Rohlf FJ, Sokal RR (1995). Statistical tables 3rd edition.

[23] Janovy J, Clopton RE, Clopton DA, Snyder SD, Efting A, Krebs L (1995). Species density distributions as null models for ecologically significant interactions of parasite species in an assemblage. Ecol Model.

[24] Khanal LK, Choudhury DR, Rai SK, Sapkota J, Barakoti A, Amatya R (2011). Prevalence of intestinal worm infestations among school children in Kathmandu, Nepal. Nepal Med Coll J.

[25] Sayasone S, Mak TK, Vanmany M, Rasphone O, Vounatsou P, Utzinger J (2011). Helminth and intestinal protozoa infections, multiparasitism and risk factors in champasack province Lao People’s democratic republic. PLoS Negl Trop Dis.

[26] Wang LC (2004). Changing patterns in intestinal parasitic infections among Southeast Asian laborers in Taiwan. Parasitol Res.

[27] Nematian J, Nematian E, Gholamrezanezhad A, Asgari AA (2004). Prevalence of intestinal parasitic infections and their relation with socio-economic factors and hygienic habits in Tehran primary school students. Acta Trop.

[28] Steinmann P, Zhou XN, Li YL, Li HJ, Chen SR, Yang Z (2007). Helminth infections and risk factor analysis among residents in eryuan county, Yunnan province, China. Acta Trop.

[29] Utzinger J, Becker SL, Knopp S, Blum J, Neumayr AL, Keiser J (2012). Neglected tropical diseases: diagnosis, clinical management, treatment and control. Swiss Med Wkly.

[30] Abu-Madi MA, Behnke JM, Prabhaker KS, Al-Ibrahim R, Lewis JW (2010). Intestinal helminths of feral cat populations from urban and suburban districts of Qatar. Vet Parasitol.

[31] Behnke JM, De Clercq D, Sacko M, Gilbert FS, Ouattara DB, Vercruysse J (2000). The epidemiology of human hookworm infections in the Southern Region of Mali West Africa. Trop Med Int Health.

[32] De Clercq D, Sacko M, Behnke J, Gilbert F, Dorny P, Vercruysse J (1997). Failure of mebendazole in treatment of human hookworm infections in the Southern Region of Mali. Am J Trop Med Hyg.

[33] Sacko M, De Clercq D, Behnke JM, Gilbert FS, Dorny P, Vercruysse J (1999). Comparison of the efficacy of mebendazole, albendazole and pyrantel in treatment of human hookworm infections in the Southern Region of Mali, West Africa. Trans Roy Soc Trop Med Hyg.

[34] Savioli L, Albonico M, Engels D, Montresor A (2004). Progress in the prevention and control of schistosomiasis and soil-transmitted helminthiasis. Parasitol Int.

[35] Albonico M, Allen H, Chitsulo L, Engels D, Gabrielli AF, Savioli L (2008). Controlling soil-transmitted helminthiasis in pre-school-age children through preventive chemotherapy. PLoS Negl Trop Dis.

[36] Anderson R, Truscott J, Hollingsworth TD. The coverage and frequency of mass drug administration required to eliminate persistent transmission of soil-transmitted helminths doi:10.1098/rstb.2013.0435#_blank%23Digital Object Identifier.Philos Trans R Soc Lond B Biol Sci. 2014;369: ISSN: 0962–8436. 10.1098/rstb.2013.0435 PMC4024228 24821921

[37] Boatin BA, Basáñez MG, Prichard RK, Awadzi K, Barakat RM (2012). A research agenda for helminth diseases of humans: towards control and elimination. PLoS Negl Trop Dis.

[38] WHO (2015). Soil-transmitted helminthiases: number of children treated in 2013. Wkly Epidemiol Rec.

